# The relationship between training intensity and training effort: the chain mediation of cognitive exhaustion and emotional blunting

**DOI:** 10.3389/fpsyg.2026.1829809

**Published:** 2026-06-02

**Authors:** Mengyuan Kong, Zhongjun Chen, Yuanyuan Hao

**Affiliations:** 1 College of Physical Education and Health Management, Henan Finance University, Zhengzhou, China; 2 School of Physical Education and Health Sciences, Guangxi Science & Technology Normal University, Changzhou, China; 3 School of Economics, Jiangsu University of Technology, Changzhou, Jiangsu, China

**Keywords:** cognitive exhaustion, emotional blunting, psychological transmission mechanism, training commitment, training control intensity

## Abstract

**Introduction:**

Training control is an important component of competitive training management, but excessive control intensity may impose psychological costs on athletes and weaken their sustained engagement in training. Drawing on Self-Determination Theory, Self-Regulatory Resource Theory, and Emotion Processing Theory, this study examined the relational mechanism linking training control intensity, cognitive exhaustion, emotional blunting, and training engagement.

**Methods:**

Cross-sectional questionnaire data were collected from 768 athletes. Confirmatory factor analysis, structural equation modeling, and multigroup analysis were used to test the proposed chain mediation model and examine whether the hypothesized pathways varied across gender and sport type.

**Results:**

Training control intensity was positively associated with cognitive exhaustion, which was further associated with higher levels of emotional blunting. Emotional blunting was negatively associated with training engagement. Cognitive exhaustion and emotional blunting together formed a significant chain-mediated pathway between training control intensity and training engagement. Multigroup analyses showed that gender moderated the path from training control intensity to cognitive exhaustion, whereas sport type moderated the paths from cognitive exhaustion to emotional blunting and from emotional blunting to training engagement.

**Discussion:**

These findings suggest that training control should be understood not only in terms of management efficiency but also in relation to athletes' psychological resource depletion and emotional responsiveness. Excessive control intensity may undermine training engagement through a sequential process involving cognitive exhaustion and emotional blunting. In practice, coaches should avoid overly frequent corrective control, provide clearer explanatory feedback, and remain attentive to athletes' cognitive and emotional states in high-intensity training contexts.

## Introduction

As competitive sports training management moves beyond outcome-oriented and load-accumulation models toward a more refined paradigm that emphasizes process quality, athlete sustainability, and psychological resilience, training control has become an important contextual factor in explaining athletes' training engagement. In high-intensity, high-density, and evaluation-driven training systems, training control is not limited to technical instruction or workload regulation. It is also embedded in training tempo, feedback frequency, behavioral norms, and punishment expectations, all of which may shape athletes' cognitive load and emotional experiences during training (Sarkar and Fletcher, 2014; Bartholomew et al., 2009). Therefore, maintaining training standards while reducing the psychological resource depletion and engagement decline associated with excessive control intensity has become a key concern in the modernization of training management.

Research grounded in Self-Determination Theory (SDT), Self-Regulatory Resource Theory, and athlete burnout has shown that highly controlling, closely monitored, and low-autonomy training environments may be associated with stronger controlled motivation, greater psychological resource depletion, and poorer training experience quality (Inzlicht and Schmeichel, 2012; Gustafsson et al., 2018). However, in competitive training practice, control is still commonly regarded as a necessary means of maintaining discipline, ensuring compliance, and improving performance. Its psychological costs may therefore be concealed by visible performance outcomes and outward behavioral conformity. This problem is especially evident in highly standardized training systems. Athletes may appear behaviorally engaged while simultaneously experiencing cognitive depletion caused by continuous self-monitoring, repeated behavioral adjustment, and emotional suppression. Such depletion may further weaken their emotional responsiveness to training and reduce their sustained engagement.

Existing sport research has largely explained the consequences of controlling training experiences through separate pathways, including motivation type, training load, fatigue, and burnout. Less attention has been given to the sequential psychological processes through which training control intensity may relate to training engagement. First, few studies have conceptualized training control intensity as a variable feature of training governance, which limits understanding of how the frequency and density of control demands are associated with athletes' psychological resource states. Second, proximal emotional mechanisms, such as emotional blunting and emotional desensitization, remain insufficiently examined. As a result, the process through which cognitive exhaustion may lead to emotional deactivation and, ultimately, lower training engagement has not been fully clarified. More direct empirical evidence is therefore needed to examine the sequential pathway linking training control intensity, cognitive exhaustion, emotional blunting, and training engagement in competitive training contexts.

Based on these theoretical and practical considerations, this study focuses on training control intensity as a key situational variable and constructs a chain mediation model of “training control intensity—cognitive exhaustion—emotional blunting—training engagement.” Using cross-sectional questionnaire data from athletes and structural equation modeling, the study examines the direct and indirect associations among these variables and further tests whether gender and sport type moderate key pathways. Given the cross-sectional design, the findings are intended to reveal statistical associations and theoretically informed explanatory pathways rather than establish strict directional causality. This study seeks to advance understanding of the psychological costs embedded in high-control training contexts and to provide empirical evidence for balancing training standards with athletes' psychological sustainability.

## Theoretical foundations and research hypotheses

### Theoretical foundations

#### Controlled training contexts and the generative logic of engagement motivation (self-determination theory)

Self-determination theory (SDT), developed by Deci and Ryan within motivational psychology, argues that behavioral quality depends less on motivational intensity than on the degree of internalization of motivational regulation. SDT identifies autonomy, competence, and relatedness as fundamental psychological needs that support self-endorsed, high-quality motivation. When social contexts cultivate choice, efficacy, and relational security, individuals are more likely to regulate behavior autonomously, which is reflected in sustained engagement and persistence (Ryan and Deci, 2000, 2017).

In sport and training settings, SDT is widely used to explain how coaching styles shape athletes' motivational regulation. Relative to autonomy-supportive practices, controlling training climates maintain behavioral order through directive communication, unilateral decision-making, intensive monitoring, and the anticipation of punishment. Their underlying logic prioritizes compliance, efficiency, and outcome standardization. By narrowing the space for choice, weakening athletes' interpretive agency, and reducing perceived self-causality, such climates shift regulation away from self-determined motives and toward external contingencies. Empirical evidence indicates that highly controlling interaction patterns increase athletes' propensity for controlled motivation: although short-term execution may remain high, engagement is less likely to consolidate into stable, self-consistent long-term commitment (Ntoumanis, 2001).

Importantly, the motivational impact of controlling climates is rarely confined to overt resistance or immediate declines in drive. More commonly, it operates through the chronic frustration of basic psychological needs. Frequent directive interventions and outcome-centered evaluations diminish athletes' perceived autonomy, externalizing the meaning of training to imposed standards and requirements. An overemphasis on error correction and social comparison can undermine perceived competence, shifting the training experience toward defensiveness and risk avoidance (Stebbings et al., 2011). Meanwhile, interactions that provide limited emotional responsiveness and little interpretive space may erode relatedness, gradually reframing training as depersonalized task execution. Under these conditions, athletes may continue to participate behaviorally, yet their engagement increasingly reflects passive compliance rather than active investment (Bartholomew et al., 2009).

Taken together, SDT provides a rigorous framework for conceptualizing motivational vulnerabilities in controlled training contexts. It highlights the structural motivational costs of control—namely, how intensified control can reconfigure the regulatory basis of training behavior by constraining pathways for psychological need satisfaction. This perspective establishes the motivational foundation for examining how control intensity undermines training engagement through downstream cognitive and affective mechanisms. It also offers a coherent theoretical bridge to the proposed sequential process involving resource depletion, affective blunting, and reduced behavioral persistence.

#### Continuous self-regulation load and the resource mechanism of cognitive exhaustion (self-regulation resource theory)

Self-Regulatory Resource Theory, advanced by Baumeister and colleagues in foundational work on self-control, posits that sustained engagement in regulatory acts—such as emotion suppression, behavioral inhibition, and attentional control—progressively consumes finite psychological resources. As these resources are depleted, individuals experience a transient reduction in cognitive efficiency and volitional control, a state commonly described as self-depletion or cognitive exhaustion. From this perspective, self-control functions as a limited-capacity energy system: overexertion compromises subsequent concentration, decision quality, and emotion regulation (Baumeister et al., 2018).

In competitive training, self-regulation is not episodic but routinized as a normative psychological requirement. Athletes are expected to suppress fatigue cues, inhibit negative affect, sustain task-focused attention, and continually recalibrate performance under dense feedback and evaluative scrutiny. Such high-frequency regulatory effort renders the training environment a primary context for cumulative depletion of self-regulatory resources (Inzlicht and Schmeichel, 2012). Controlling climates further intensify this burden by increasing normative constraints and evaluative density, thereby compelling continuous self-monitoring and behavioral adjustment. Athletes must repeatedly appraise their alignment with coaching expectations while inhibiting and modulating emotional reactions, verbal expressions, and action tendencies—processes that draw heavily on self-regulatory capacity. When these demands recur across training cycles, the resources available for cognitive processing and affect management progressively erode, manifesting as attentional instability, slowed decision-making, and weakened emotion regulation. These functional impairments signal the onset of cognitive exhaustion (Muraven and Baumeister, 2000).

In sum, Self-Regulatory Resource Theory explains how controlling environments accelerate psychological resource depletion by chronically elevating regulatory demands, thereby precipitating cognitive exhaustion. In the present study, this framework substantiates the proposed pathway from training control intensity to cognitive exhaustion and provides a mechanistic bridge for examining how exhaustion subsequently reshapes affective responding and, ultimately, training commitment.

#### Emotional reactivity dulling and the affective channel of training engagement decline (emotion processing theory)

Affective processing theory, originating in Lazarus's appraisal framework, conceptualizes emotion as the product of individuals' cognitive evaluations of situational threat, controllability, and personal significance. When contexts are chronically characterized by high stress and limited reward, appraisal processes adapt over time, systematically recalibrating emotional intensity and arousal (Lazarus and Folkman, 1984).

In persistently demanding environments, affective adaptation does not necessarily manifest as overt negative emotion. Rather than sustained high-arousal states such as anxiety or anger, individuals may develop blunted affective reactivity, characterized by low arousal and reduced responsiveness (Litz and Gray, 2002). This phenomenon is particularly relevant to competitive training. Prolonged exposure to dense evaluation, frequent error correction, and continuous performance pressure compels athletes to repeatedly regulate emotional reactions in order to maintain stable and controllable outward behavior. As suppression and regulation become routinized, the affective system may progressively downregulate sensitivity to training-related cues. Consequently, experiences that once elicited accomplishment, challenge, or interest may be appraised as emotionally neutral, thereby reframing training as depersonalized task execution (Litz and Gray, 2002). Notably, this process need not culminate in explicit aversion or withdrawal; instead, it may unfold through a sustained reduction in arousal that gradually diminishes the capacity to derive affective feedback and meaning from training.

Mechanistically, cognitive depletion may accelerate the emergence of affective blunting. When self-regulatory resources are exhausted, the capacity for elaborated appraisal, emotion differentiation, and meaning-making is constrained. Affective responding then becomes simplified and compressed, reflected in attenuated emotional intensity and a flattened subjective experience (Inzlicht and Schmeichel, 2012). Under such conditions, positive affective signals in training are less likely to be fully processed and consolidated. Negative affect may not intensify overtly, yet it can persist in muted forms such as numbness or insensitivity, contributing to a broader tendency toward emotional deactivation (Frewen and Lanius, 2006).

In sum, emotion-processing perspectives suggest that sustained high-stress environments with limited emotional reinforcement can reorganize appraisal and response patterns, thereby fostering emotional blunting. This framework provides an affective rationale for the proposed pathway from cognitive depletion to emotional blunting and offers a theoretical basis for explaining how altered emotional functioning may translate into diminished training engagement.

### Research hypotheses

Based on the theoretical framework outlined above, this study defines training control intensity as the density of control demands embedded in the training context, including prescriptive rules, external monitoring, corrective pressure, and punishment expectations. It is regarded as a key contextual variable for explaining differences in athletes' training engagement. Theoretically, higher training control intensity may impose greater self-regulatory demands and may be associated with cognitive resource depletion, emotional desensitization, and reduced engagement. Accordingly, this study constructs a chain mediation model of “training control intensity—cognitive exhaustion—emotional blunting—training engagement” and incorporates gender and sport type as sources of contextual heterogeneity, thereby forming a testable system of conditional process hypotheses.

H1: Relationship between Training Control Intensity and Cognitive Exhaustion

Training interactions characterized by sustained high control may restrict athletes' autonomous interpretation of training tasks and intensify demands for behavioral adjustment and emotional suppression. Under such conditions, athletes are required to engage more frequently in attention maintenance, conflict inhibition, and self-monitoring, all of which consume substantial self-regulatory resources. Therefore, training control intensity may be positively associated with cognitive exhaustion (Inzlicht and Schmeichel, 2012).

H1a: Higher training control intensity is associated with higher levels of cognitive exhaustion among athletes.

H1b: Gender moderates this relationship; the positive association between training control intensity and cognitive exhaustion is stronger among female athletes.

H2: Relationship between Cognitive Exhaustion and Emotional Blunting.

Cognitive exhaustion reflects a reduction in the psychological resources available for nuanced emotional processing and meaning construction. When cognitive resources are depleted, athletes may process emotional cues in training in a more compressed and attenuated manner. This may manifest as emotional blunting, characterized by reduced emotional reactivity, flattened emotional experience, and diminished positive emotional feedback (Litz and Gray, 2002).

H2a: Higher levels of cognitive exhaustion are associated with higher levels of emotional blunting among athletes.

H2b: Sport type moderates this relationship; compared with team sports, the positive association between cognitive exhaustion and emotional blunting is stronger in individual sports.

H3: Relationship between Emotional Blunting and Training Engagement.

Training engagement depends partly on emotional feedback and intrinsic motivational experience during training. Higher emotional blunting may indicate that athletes have greater difficulty deriving positive arousal, affective reinforcement, and perceived meaning from training activities. As a result, training may be experienced as emotionally neutral, monotonous, or psychologically hollow, which may weaken sustained engagement. Therefore, emotional blunting may be negatively associated with training engagement (Frewen and Lanius, 2006).

H3a: Higher levels of emotional blunting are associated with lower levels of training engagement among athletes.

H3b: Sport type moderates this relationship; compared with team sports, the negative association between emotional blunting and training engagement is stronger in individual sports.

H4: Chain Mediation Effect of Training Control Intensity, Cognitive Exhaustion, and Emotional Blunting.

The relationship between training control intensity and training engagement may unfold through a sequential psychological process. Higher control intensity may be associated with greater cognitive exhaustion, which may further contribute to the deactivation of emotional responses and ultimately relate to lower training engagement (Muraven and Baumeister, 2000).

H4: Cognitive exhaustion and emotional blunting constitute a chained mediating pathway between training control intensity and training engagement: training control intensity → cognitive exhaustion → emotional blunting → training engagement.

H5: Conditional Process Effects of Gender and Sport Type.

If key psychological transition points are shaped by stable contextual heterogeneity, the indirect association between training control intensity and training engagement may vary across athlete groups. Specifically, the strength of the chained mediation effect may differ by gender and sport type (Stebbings et al., 2011).

H5a: Gender moderates the indirect association between training control intensity and training engagement through cognitive exhaustion; this indirect association is stronger among female athletes.

H5b: Sport type moderates the chained mediating association between training control intensity and training engagement through the pathway of cognitive exhaustion → emotional blunting; this indirect association is stronger in individual sports and weaker in team sports.

**Figure 1:**
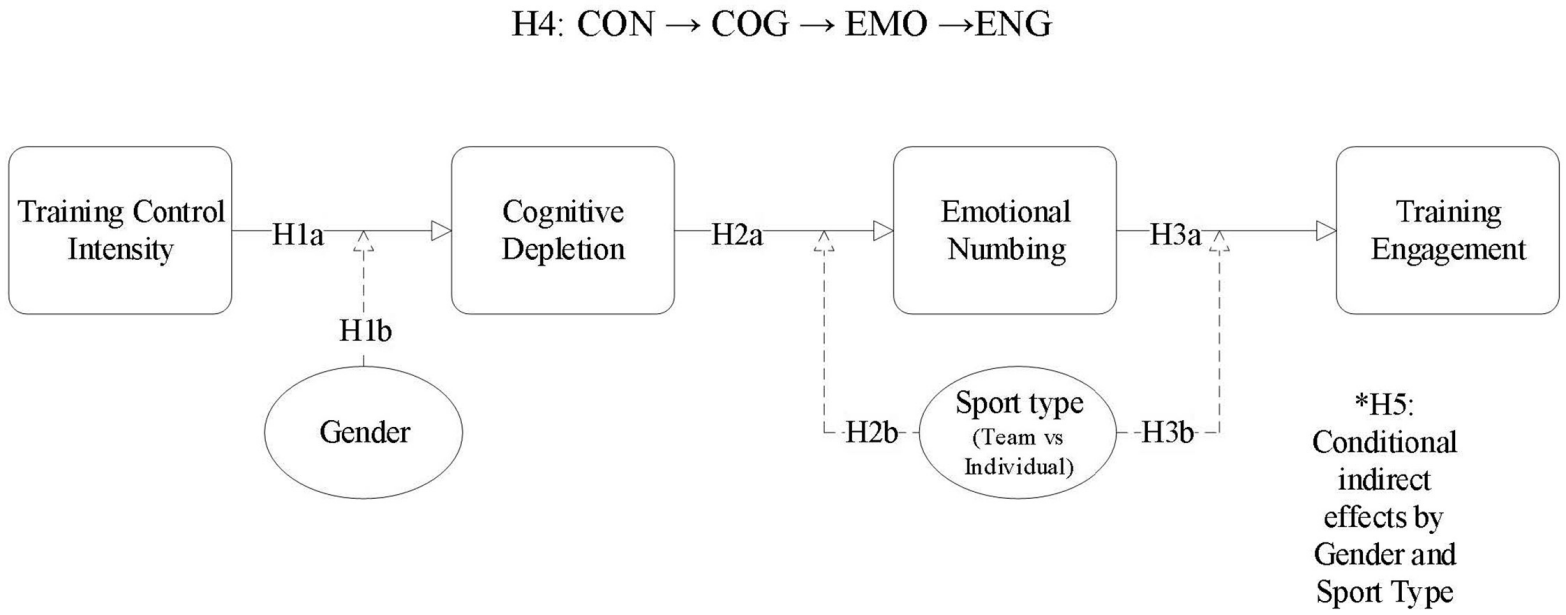
Conceptual model of the study. *Note* training control intensity = CON; cognitive depletion = COG; emotional numbness = EMO; training engagement = ENG.

In summary, the hypothesized model tested in this study is presented in Figure 1.

## Research design

### Study population and sample sources

This study adopted a multistage stratified cluster sampling design. The target population comprised active athletes from university competitive training programs and provincial training units in China. A questionnaire survey was conducted to examine the chained mediating mechanism linking training control intensity, cognitive exhaustion, emotional blunting, and training engagement. Gender and sport type were included as sources of contextual heterogeneity to support subsequent multigroup analyses.

Participants were recruited from four broad regions: North China, East China, Central China, and Southwest China. Ten provincial-level administrative regions were selected as sampling sites, including Beijing, Jiangsu, Hunan, Hubei, Sichuan, Guangdong, Shandong, Liaoning, Shaanxi, and Zhejiang. The sampling process prioritized high-level university sports teams, specialized teams at sports universities, and provincial training teams with stable training structures, explicit training regulations, and relatively standardized evaluation procedures. Data collection was conducted at the team or sport-specialty level, with each team or specialty treated as a cluster unit.

The selection of university competitive training programs and provincial training units was based on two considerations. First, these organizations generally operate under hierarchical management, centralized training arrangements, and performance evaluation systems, which provide a suitable context for observing training control intensity. Second, their relatively consistent institutional and cultural backgrounds help reduce interference from cross-institutional variation, thereby allowing a more focused examination of the association between control intensity and athletes' psychological states within standardized training settings. Nevertheless, this sampling strategy also requires caution when extending the findings to different cultural or institutional contexts.

During data collection, trained researchers distributed and collected the questionnaires on site at the participating training units. Participants completed the questionnaires anonymously and were informed that the data would be used exclusively for academic research and treated as strictly confidential. These procedures were implemented to reduce evaluation anxiety and social desirability bias. A total of 860 questionnaires were distributed, of which 823 were returned. After excluding invalid responses due to excessive missing data, uniform response patterns, or markedly abnormal completion times, 768 valid questionnaires were retained. The invalid response rate was 6.7%, and the valid response rate was 89.3%.

This study was approved by the Academic Ethics Committee of Jiangsu Institute of Technology before data collection (Approval No.: JUT-2025-025). All participants provided voluntary informed consent after being informed of the study objectives, data use, anonymization procedures, and their right to withdraw. The research process complied with the Declaration of Helsinki and relevant ethical guidelines. All questionnaire data were used solely for academic research and were anonymized during statistical analysis to protect participants' confidentiality. For participants younger than 18 years, informed consent was obtained from both the participant and a parent or legal guardian.

Participants ranged in age from 16 to 24 years (*M* = 19.7, *SD* = 1.9), and their training experience ranged from 3 to 13 years (*M* = 7.1, *SD* = 2.6). The sample included 432 male athletes (56.3%) and 336 female athletes (43.7%), which met the requirements for gender-based comparative analysis. Sport type was classified according to training organization structure. A total of 402 athletes (52.3%) participated in team sports, including basketball, soccer, volleyball, and handball, whereas 366 athletes (47.7%) participated in individual sports, including track and field, swimming, gymnastics, wushu routines, and taekwondo. This distribution provided an adequate basis for subsequent comparisons using multigroup structural models.

In terms of athletic ranking, 126 participants were National Class 1 athletes (16.4%), 356 were National Class 2 athletes (46.4%), and 286 had no formal ranking or held only a school-level ranking (37.2%). Regarding training systems, athletes from university training programs accounted for approximately 62.5% of the sample, while athletes from provincial training units accounted for approximately 37.5%. This composition preserved a degree of training management heterogeneity while maintaining a coherent sampling framework.

### Measurement instruments

This study employed a single-source, athlete self-report questionnaire system grounded in the theoretical pathway training control intensity → cognitive exhaustion → emotional blunting → training commitment. This design ensured clear operationalization of latent constructs and conceptual coherence across hypothesized pathways. All measures were completed independently by athletes to minimize systematic biases commonly associated with multi-source designs, particularly discrepancies arising from temporal misalignment and differences in contextual perception. Accordingly, the protocol aimed to capture athletes' subjective experiential structures in training contexts with greater fidelity.

#### Training control intensity

Training control intensity assessed athletes' perceived density of directive rules, degree of external monitoring, pressure from error correction, and severity of anticipated punishment during routine training. The scale was contextually adapted from the Controlling Coaching Behavior Scale (Bartholomew et al., 2009) and consisted of eight items, including: “I have little room to autonomously adjust training methods,” “Coaches frequently provide high-frequency error corrections focused on performance outcomes,” and “Failure to meet performance standards results in explicit punishment.”

Responses were recorded on a five-point Likert scale (1 = strongly disagree, 5 = strongly agree), with item means aggregated to form the scale score. Preliminary analyses demonstrated excellent internal consistency (Cronbach's α = 0.96), high composite reliability (*CR* = 0.97), and strong convergent validity (*AVE* = 0.80). Confirmatory factor analysis (CFA) indicated standardized factor loadings ranging from 0.84 to 0.93, all statistically significant (*p* < 0.001).

#### Cognitive exhaustion

Cognitive exhaustion measured athletes' perceived depletion of psychological resources under sustained high-demand training conditions. The scale was developed based on the self-regulatory resource model (Inzlicht and Schmeichel, 2012), incorporating classic self-control depletion indicators from Muraven and Baumeister (2000). It comprised six items, such as: “After training, I find it difficult to maintain focus,” “Even simple training tasks leave me feeling mentally exhausted,” and “I feel that my mental energy is depleted by training.”

Items were rated on a five-point Likert scale (1 = strongly disagree, 5 = strongly agree), with mean scores representing overall cognitive exhaustion. Reliability and validity indices were satisfactory (α = 0.93, *CR* = 0.95, *AVE* = 0.76), and standardized factor loadings ranged from 0.82 to 0.90 (*p* < 0.001).

#### Emotional blunting

Emotional blunting assessed reduced emotional reactivity and flattened affective experience during training. Drawing on theoretical conceptualizations of emotional numbing (Litz and Gray, 2002; Frewen and Lanius, 2006), the scale included five items adapted to competitive training contexts, such as: “It is difficult to feel excited or anticipatory during training,” “My emotional reactions remain minimal regardless of performance outcomes,” “My sense of accomplishment during training has gradually diminished,” and “I increasingly feel detached from my emotions.”

Responses were recorded on a five-point Likert scale (1 = strongly disagree, 5 = strongly agree), with item means calculated as the total score. The scale demonstrated strong internal consistency (α = 0.91), high composite reliability (*CR* = 0.93), and satisfactory convergent validity (*AVE* = 0.74). Standardized factor loadings ranged from 0.84 to 0.93 (*p* < 0.001).

#### Training commitment

Training commitment captured athletes' sustained focus, effort investment, and persistence during training. The scale was adapted from the Utrecht Work Engagement Scale (Schaufeli et al., 2002) for competitive training contexts and comprised six items, including: “I maintain high levels of concentration during training,” “I am willing to give my all in training even when tired,” and “I proactively make extra efforts to improve my performance.”

Items were rated on a five-point Likert scale (1 = strongly disagree, 5 = strongly agree), with mean scores representing overall training commitment. Preliminary analyses indicated excellent reliability (α = 0.94, *CR* = 0.96, *AVE* = 0.80), and standardized factor loadings ranged from 0.86 to 0.93 (*p* < 0.001). Both convergent and discriminant validity met established requirements for structural equation modeling.

All instruments employed a five-point Likert response format to ensure metric comparability across latent constructs. During the pilot phase, 150 athletes representing both team and individual sports participated in pretesting. Psychometric evaluation indicated satisfactory measurement quality: all scales exhibited Cronbach's α > 0.80, composite reliability (CR) > 0.85, and average variance extracted (AVE) > 0.50, meeting established thresholds for subsequent structural equation modeling.

To strengthen alignment among construct definitions, item wording, and the competitive training context, the questionnaire underwent a two-stage semantic refinement process. Two doctoral researchers with expertise in sport psychology independently reviewed each item for conceptual correspondence and linguistic clarity. Their revisions minimized ambiguity and improved interpretability, and pilot feedback indicated no substantive comprehension difficulties. Collectively, these procedures provided a robust measurement foundation for formal data collection.

### Data analysis methods

Data analysis and model testing were conducted using SPSS 26.0 and AMOS 24.0. Before estimating the structural model, the distributional properties of the sample data were examined to determine their suitability for maximum likelihood estimation. In the pilot sample, the absolute skewness values of all core variables were below 1.10, and the absolute kurtosis values were below 2.40. These results indicated no substantial deviation from normality and satisfied the basic distributional assumptions for structural equation modeling. Correlation analyses were then performed among training control intensity, cognitive exhaustion, emotional blunting, and training engagement. The observed associations were generally consistent with theoretical expectations, thereby providing an empirical basis for subsequent path analysis. Given the use of single-time-point, cross-sectional, self-report data, the structural equation model was employed primarily to examine model fit, path associations, and indirect effects among variables. It was not used to establish temporal ordering or strict causal relationships.

A structural equation model was subsequently constructed to test the proposed pathway of training control intensity → cognitive exhaustion → emotional blunting → training engagement. The model simultaneously estimated direct and indirect associations, with particular emphasis on whether training control intensity was indirectly associated with training engagement through cognitive exhaustion and emotional blunting. During model estimation, confirmatory factor analysis was first conducted to assess the construct validity of the latent variables. After the measurement model reached acceptable fit, the structural paths were estimated and interpreted. The preliminary measurement model demonstrated good overall fit: χ^2^/df = 2.41, *CFI* = 0.94, *TLI* = 0.93, *RMSEA* = 0.043, and *SRMR* = 0.052. These indices suggested that the observed indicators adequately represented their corresponding latent constructs.

At the structural level, the analysis examined the direct association between training control intensity and training engagement, as well as the indirect pathways mediated by cognitive exhaustion and emotional blunting. To avoid the limitations of traditional stepwise regression in mediation testing, a non-parametric bootstrap procedure with 5,000 resamples was used to estimate indirect effects. Mediation effects were evaluated using 95% confidence intervals. The preliminary results showed that cognitive exhaustion and emotional blunting formed a significant chained mediating pathway between training control intensity and training engagement. The estimated indirect effect was −0.21, with a 95% confidence interval of [−0.29, −0.14], which did not include zero. This result indicates that the association between training control intensity and training engagement may be partially explained by a sequential process involving psychological resource depletion and emotional response deactivation.

Model fit was evaluated using both absolute and incremental fit indices, including χ^2^/df, CFI, TLI, RMSEA, and SRMR. Overall, the structural model showed good fit in the pilot sample, with no evidence of substantial model misfit.

To examine the structural stability of the proposed psychological mechanism across different training contexts, multigroup structural equation modeling was conducted using gender and sport type as grouping variables. Sport type was classified into team sports and individual sports according to the organizational structure of training. An unconstrained baseline model was estimated first. Equality constraints were then imposed sequentially on key structural paths, and changes in model fit were compared to identify potential between-group differences. A ΔCFI value below 0.01 was used as the primary criterion for structural equivalence, with Δχ^2^ used as a supplementary test of specific path differences. The preliminary results indicated that the path from cognitive exhaustion to emotional blunting differed significantly across sport types (Δχ^2^ = 5.12, *p* < 0.05), whereas the remaining paths were relatively stable across gender groups. This suggests that the strength of the chained mechanism may vary by training organization structure, while the overall structural framework remains largely consistent.

These analytical procedures were conducted during the pilot phase to evaluate the model's discriminative capacity, path stability, and feasibility for multigroup comparison. The results provided methodological support and parameter references for the subsequent analysis of the main sample.

## Research findings

### Descriptive statistics and correlation analysis

To characterize the distributions and bivariate associations among the focal constructs, we first computed descriptive statistics and Pearson correlations for training control intensity, cognitive exhaustion, emotional blunting, and training commitment.

As shown in Table 1, the means clustered near the midpoint of the scale (*M* = 2.99–3.01), with standard deviations ranging from 1.11 to 1.14. This pattern indicates comparable central tendencies across constructs while retaining adequate variability, thereby supporting subsequent covariance-based path estimation.

**Table 1:** Descriptive statistics.

Variable	Mean	Standard deviation
Training control intensity (CON)	3.00	1.13
Cognitive depletion (COG)	3.00	1.11
Emotional numbness (EMO)	3.00	1.11
Training engagement (ENG)	3.00	1.14

**Figure 2:**
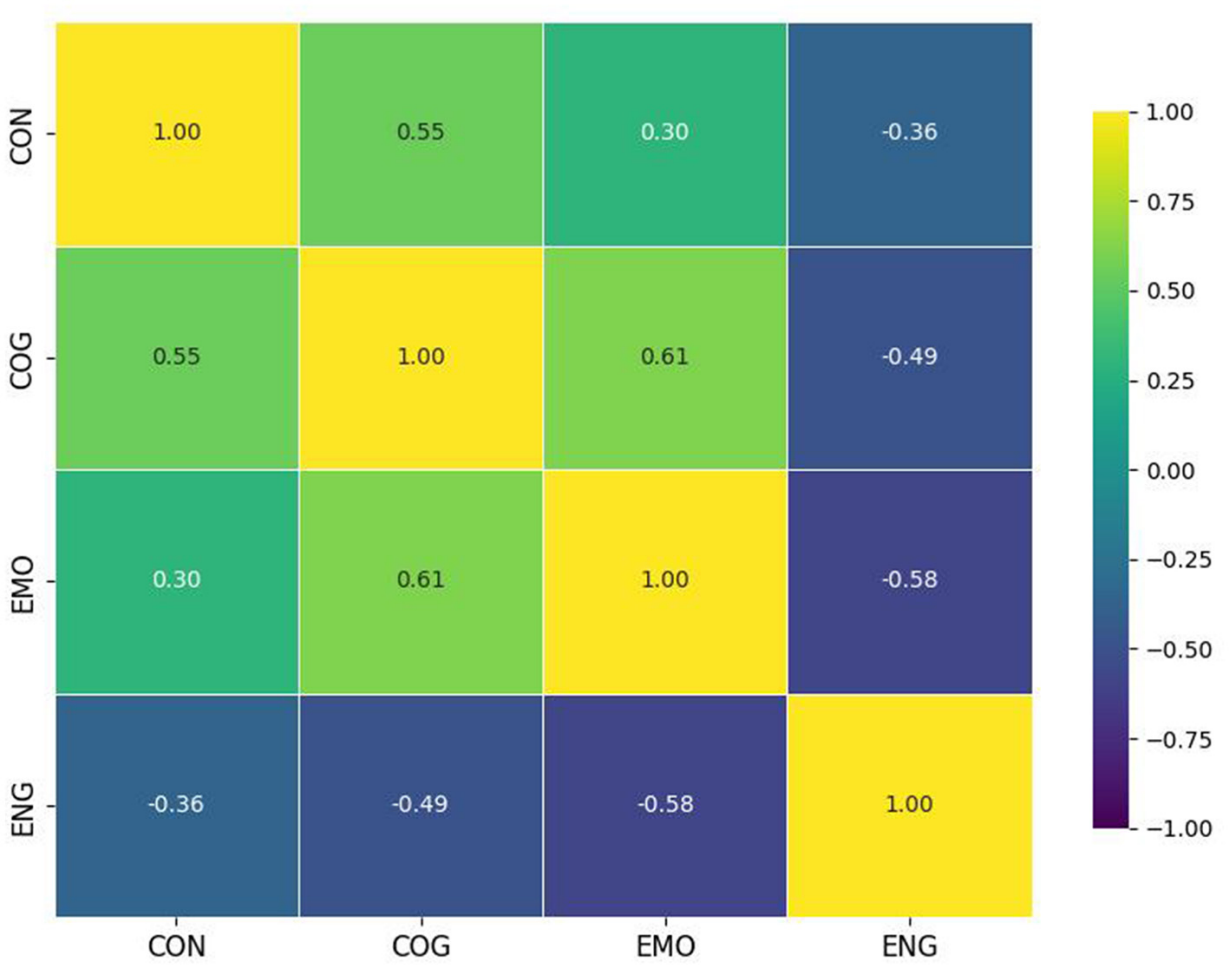
Pearson correlation matrix among core variables.

Figure 2 presents the Pearson correlation matrix for the core variables. The overall pattern of associations was consistent with the theoretical expectations of this study. Training control intensity was moderately and positively correlated with cognitive exhaustion (*r* = 0.55), suggesting that athletes in highly controlled training environments tended to experience greater cognitive resource depletion. Cognitive exhaustion was positively correlated with emotional blunting (*r* = 0.61), indicating that higher levels of cognitive exhaustion were accompanied by stronger emotional attenuation. Emotional blunting was negatively correlated with training engagement (*r* = −0.58), suggesting that reduced emotional responsiveness was associated with lower engagement in training.

Training control intensity was also negatively correlated with training engagement (*r* = −0.36), indicating that stronger control demands were associated with lower levels of engagement. Similarly, cognitive exhaustion was negatively correlated with training engagement (*r* = −0.49), suggesting that cognitive resource depletion was accompanied by a decline in athletes' training engagement.

Taken together, the correlation results among the core variables were broadly consistent with the proposed theoretical model. These findings provide preliminary empirical evidence for subsequent tests of structural paths and mediating effects using structural equation modeling.

### Measurement model validation: confirmatory factor analysis (CFA)

Because all core variables were assessed using athlete self-report questionnaires, this study first conducted Harman's single-factor test to evaluate potential common method bias. All items measuring training control intensity, cognitive exhaustion, emotional blunting, and training engagement were entered into an unrotated exploratory factor analysis. The results showed that the first factor explained 31.84% of the total variance, which was below the commonly accepted 40% threshold. This finding indicates that common method bias was unlikely to pose a substantial threat to the validity of the results. To further assess this issue, a one-factor confirmatory factor analysis was performed. The one-factor model showed poor fit to the data: χ^2^/df = 9.27, *CFI* = 0.62, *TLI* = 0.58, *RMSEA* = 0.10, and *SRMR* = 0.09. Its fit was markedly inferior to that of the four-factor measurement model. These results suggest that the primary measurement variance could not be adequately explained by a single latent factor. Therefore, common method bias was unlikely to account for the observed associations among training control intensity, cognitive exhaustion, emotional blunting, and training engagement.

To verify the factorial structure of the study constructs, confirmatory factor analysis (CFA) was conducted to evaluate the reliability and validity of the measurement model. Table 2 reports internal consistency (Cronbach's α), composite reliability (CR), and average variance extracted (AVE) for the four latent variables.

**Table 2:** Confirmatory factor analysis results.

Latent variable	Cronbach α	CR	AVE
CON	0.96	0.97	0.79
COG	0.93	0.95	0.76
EMO	0.91	0.93	0.74
ENG	0.94	0.96	0.80

As shown in Table 2, all constructs demonstrated strong internal consistency, with Cronbach's α values exceeding 0.90, thereby surpassing the conventional 0.80 criterion. In particular, training control intensity (CON) and training commitment (ENG) exhibited α values of 0.95 and 0.94, respectively, indicating highly coherent item sets. Consistent with these results, CR values ranged from 0.93 to 0.97, well above the 0.70 benchmark, suggesting strong construct reliability and substantial shared variance between each latent factor and its indicators.

Convergent validity was further supported by AVE values above 0.50 for all constructs (*CON* = 0.79, *COG* = 0.76, *EMO* = 0.74, *ENG* = 0.80), indicating that the latent variables accounted for more variance in their indicators than was attributable to measurement error.

Table 3 summarizes the measurement model fit indices. The model demonstrated good fit, with χ^2^/df = 2.85, *CFI* = 0.96, and *TLI* = 0.95, meeting or exceeding recommended thresholds. Approximation error indices also supported adequate fit (*RMSEA* = 0.05, *SRMR* = 0.03), indicating minimal residual misfit.

**Table 3:** Model fit indices for measurement mode.

Fit index	Model value	Recommended threshold
χ^2^/df	2.85	< 3.00
CFI	0.96	>0.90
TLI	0.95	>0.90
RMSEA	0.05	< 0.08
SRMR	0.03	< 0.08

Collectively, these findings indicate that the measurement model exhibits robust psychometric properties and satisfactory global fit, providing a sound basis for subsequent structural equation modeling. Moreover, the strong convergent performance across training control intensity, cognitive exhaustion, emotional blunting, and training commitment supports the feasibility of testing the proposed “cognitive–emotional–behavioral” sequential mechanism in the structural model.

### Structural path analysis

Building on the established reliability, convergent validity, and global fit of the measurement model, we specified a structural path model to test the proposed sequential mechanism—training control intensity → cognitive exhaustion → emotional blunting → training commitment.

**Figure 3:**
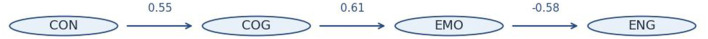
Standardized path coefficients in the SEM.

The results of the structural path analysis are presented in Figure 3. Training control intensity was significantly and positively associated with cognitive exhaustion (β = 0.55), indicating that athletes who perceived higher training control intensity tended to report greater cognitive exhaustion. This finding suggests that high-control training contexts may be closely related to increased information-processing demands, sustained attentional load, and self-regulatory burden. Cognitive exhaustion was significantly and positively associated with emotional blunting (β = 0.61), indicating that higher cognitive exhaustion was accompanied by reduced emotional responsiveness and a stronger tendency toward emotional flattening. Emotional blunting was significantly and negatively associated with training engagement (β = −0.58), suggesting that athletes with lower emotional reactivity and arousal tended to show lower levels of training focus, initiative, and sustained engagement.

To further examine the indirect associations among the variables, this study used a bootstrap procedure to test the chained mediation effect. The results showed that training control intensity was significantly and indirectly associated with training engagement through the pathway of cognitive exhaustion → emotional blunting. The indirect effect was β = −0.16, with a 95% confidence interval of [−0.201, −0.132], which did not include zero. These findings indicate that cognitive exhaustion and emotional blunting jointly functioned as chained mediators between training control intensity and training engagement. However, because the data were collected using a single-time-point cross-sectional design, the results should be interpreted as statistically significant indirect associations rather than evidence of strict causal transmission.

Regarding model explanatory power, the proposed mechanism demonstrated considerable predictive capacity. Training control intensity explained 30.4% of the variance in cognitive exhaustion (*R*^2^ = 0.30). Training control intensity and cognitive exhaustion jointly explained 37.6% of the variance in emotional blunting (*R*^2^ = 0.38). The overall structural model explained 38.7% of the variance in training engagement (*R*^2^ = 0.39). These results indicate that the proposed model was consistent with theoretical expectations in terms of path direction and showed meaningful explanatory strength at the effect-size level.

Overall, the findings support the existence of sequential associations among training control intensity, cognitive exhaustion, emotional blunting, and training engagement in high-control training contexts. Higher training control intensity was associated with greater cognitive exhaustion; cognitive exhaustion was associated with stronger emotional blunting; and emotional blunting was associated with lower training engagement. This structural evidence provides empirical support for understanding the relationship between control intensity, psychological state, and behavioral engagement within competitive training systems.

### Moderating effects analysis

After establishing the adequacy of the structural model and the significance of focal paths, we conducted multi-group structural equation modeling (SEM) to test whether gender and sport type moderated key structural relations (see Table 4). Group differences were evaluated by comparing an unconstrained baseline model with models imposing equality constraints on target paths.

**Table 4:** Multi-group SEM results.

Grouping variable	Group	Path	Standardized coefficient (β)	Δχ^2^ (df = 1)	Significance
Gender	Male	CON → COG	0.51	4.49	P = 0.03
Gender	Female	CON → COG	0.60	4.49	P = 0.03
Sport type 1	Team	COG → EMO	0.56	17.55	P < 0.001
Sport type 1	Individual	COG → EMO	0.73	17.55	P < 0.001
Sport type 2	Team	EMO → ENG	−0.39	13.32	P < 0.001
Sport type 2	Individual	EMO → ENG	−0.54	13.32	P < 0.001

Results indicated a significant moderating effect of gender on the training control intensity → cognitive exhaustion link. Relative to the freely estimated model, constraining this path to equality across gender groups produced a significant decrement in fit (Δχ^2^ (1) = 4.49, *p* = 0.034), indicating that the strength of this relation differed by gender. Consistent with this test, the standardized path coefficient was larger for the female group than for the male group (β_female = 0.60 vs. β_male = 0.51). Thus, at comparable levels of perceived control intensity, female athletes appear more susceptible to cognitive exhaustion. This pattern suggests that the resource-depleting effects of controlling training may be amplified by gender-linked differences in stress appraisal, regulatory demands, and contextual experience, increasing the likelihood of cumulative cognitive exhaustion among female athletes.

Sport type exhibited more pronounced moderation within the mediating chain. Equality constraints on the cognitive exhaustion → emotional blunting path yielded a marked loss of fit [Δχ^2^(1) = 17.55, *p* = 2.80 × 10^−5^], indicating significant differences between individual and team sports. Path estimates further showed a stronger association in individual sports (β_individual = 0.73) than in team sports (β_team = 0.56), suggesting that sustained depletion is more likely to translate into affective blunting and detachment in individual-sport contexts. A parallel pattern emerged for the emotional blunting → training commitment path, where between-group heterogeneity was also significant [Δχ^2^(1) = 13.32, *p* = 2.63 × 10^−4^]. The negative effect was stronger in individual sports (β_individual = −0.54) than in team sports (β_team = −0.39), implying that diminished affective functioning more directly undermines commitment in individual disciplines. By contrast, team settings may provide partial buffering through interpersonal interaction, shared goals, and social support, thereby attenuating the behavioral impact of emotional blunting.

Overall, the multi-group results demonstrate systematic heterogeneity in the proposed mechanism. Gender primarily moderated the entry point of the chain (training control intensity → cognitive exhaustion), whereas sport type moderated the two downstream links (cognitive exhaustion → emotional blunting and emotional blunting → training commitment). These findings indicate that the psychological and behavioral consequences of highly controlling training climates vary as a function of athlete characteristics and training ecology. Accordingly, training governance and intervention design should incorporate gender-sensitive considerations and sport-specific contextual tailoring to strengthen risk prevention and sustain training commitment.

## Discussion

### The relationship between training intensity and cognitive exhaustion: from a management tool to a resource consumption perspective

The structural model showed a significant positive association between training control intensity and cognitive exhaustion (β = 0.55). This finding indicates that athletes who perceived higher levels of training control tended to report greater psychological resource depletion. Although training control plays an important role in maintaining order, standardizing requirements, and ensuring compliance in competitive training, its potential psychological costs also warrant attention. When athletes are exposed over time to directive rules, frequent corrections, external monitoring, and punishment expectations, they must not only complete technical movements and meet workload demands but also continuously evaluate whether their performance conforms to external standards. This process requires sustained adjustment of attention, behavior, and emotional responses. Accordingly, the positive association between training control intensity and cognitive exhaustion suggests that controlling training contexts may increase athletes' allocation of psychological resources to self-monitoring and psychological regulation (Bartholomew et al., 2009; Stebbings et al., 2011).

This finding is consistent with Self-Determination Theory and Self-Regulatory Resource Theory. Self-Determination Theory emphasizes that high-quality engagement depends on the satisfaction of autonomy, competence, and relatedness needs. When training contexts place excessive emphasis on compliance, error correction, and external evaluation, athletes' autonomy in interpreting and engaging with the training process may be constrained, making their behavior more dependent on external pressure (Ryan and Deci, 2000, 2017). Self-Regulatory Resource Theory further suggests that sustained self-monitoring, conflict inhibition, and emotional control consume limited psychological resources (Muraven and Baumeister, 2000; Inzlicht and Schmeichel, 2012). In competitive training settings, high control may not immediately lead to overt resistance or withdrawal. Instead, its effects may accumulate through more subtle forms of psychological depletion, including difficulty maintaining attention, increased mental fatigue, and reduced psychological energy. Therefore, the association between training control intensity and cognitive exhaustion provides relatively direct empirical evidence for understanding psychological depletion in high-control training environments.

These results also suggest that training management should not define control solely in terms of strictness or enforcement efficiency. Moderate control remains necessary for maintaining training standards; however, when control demands accumulate continuously throughout the training process, athletes may expend substantial psychological resources to sustain outwardly stable performance. Under such conditions, control itself may become an important situational source of cognitive exhaustion. Training management should therefore distinguish necessary control from excessive control. Clear standards should be retained for safety thresholds, technical specifications, and key tactical requirements, whereas movement exploration, training feedback, and individual adjustment should allow appropriate interpretive space and autonomy.

### The relationship between cognitive exhaustion and emotional blunting: psychological resource state and emotional processing patterns

The structural model showed a significant positive association between cognitive exhaustion and emotional blunting (β = 0.61). This finding indicates that athletes with higher levels of psychological resource depletion tended to report stronger reductions in emotional reactivity and more flattened emotional experiences. Cognitive exhaustion is not limited to attentional decline, mental fatigue, or post-training tiredness; it may also be associated with reduced responsiveness in the emotional system. When athletes are exposed to prolonged high-demand training and continuous self-regulation, their limited psychological resources are more likely to be allocated to movement execution, error correction, attentional maintenance, and responses to evaluation. As a result, fewer resources remain available for emotional experience, meaning construction, and the perception of positive feedback. Under such conditions, athletes' sense of accomplishment, challenge, and anticipation during training may gradually decline, while their emotional responses to training stimuli may become increasingly attenuated.

This finding is consistent with Self-Regulatory Resource Theory and Emotion Processing Theory. Self-Regulatory Resource Theory suggests that sustained attentional control, conflict inhibition, and emotional regulation consume limited psychological resources and may impair subsequent cognitive and emotional functioning (Muraven and Baumeister, 2000; Inzlicht and Schmeichel, 2012). Emotion Processing Theory further emphasizes that emotional responses are not generated automatically; rather, they depend on individuals' ongoing appraisal of situational meaning, threat, and value (Lazarus and Folkman, 1984). When cognitive exhaustion is high, athletes may have fewer psychological resources available to process training meaning and emotional cues. Their emotional responses may therefore shift from positive arousal to reduced reactivity, limited fluctuation, and flattened experience. Existing research also indicates that, under prolonged high-stress or high-demand conditions, the emotional system may not necessarily present as intense negative emotion. Instead, it may manifest as emotional numbness, blunted responsiveness, and diminished affective experience (Litz and Gray, 2002; Frewen and Lanius, 2006).

The significance of these results lies in extending the understanding of psychological exhaustion in training from the cognitive level to the emotional level. In training practice, athletes' risk signals do not always appear as explicit complaints, anxiety, or resistance. They may instead be reflected in muted responses to training outcomes, limited emotional fluctuation in response to feedback, or diminished anticipation of challenging tasks. Such states may be misinterpreted as stability, maturity, or compliance with training arrangements. However, the findings of this study suggest that they may reflect emotional under-reactivity following cognitive exhaustion. Therefore, training management should attend not only to athletes' cognitive fatigue but also to the gradual weakening of their emotional responses. For athletes who are continuously exposed to high-control and high-evaluation environments, coaches should identify signs such as mental fatigue, flattened emotional responses, and reduced training engagement in a timely manner to prevent cognitive exhaustion from further developing into emotional blunting.

### The immediate link between emotional blunting and declining training engagement

The structural model showed a significant negative association between emotional blunting and training engagement (β = −0.58). This finding indicates that athletes with higher levels of emotional blunting, characterized by reduced emotional reactivity, tended to report lower levels of training focus, proactive effort, and sustained engagement. Training engagement does not merely refer to task completion or regular attendance; it also involves psychological components such as focused involvement, energy mobilization, and voluntary effort (Schaufeli et al., 2002). When athletes find it difficult to experience excitement, anticipation, accomplishment, or challenge during training, the training process may gradually be perceived as repetitive, monotonous, and unrewarding, even when its objectives remain clear.

This finding is consistent with Emotion Processing Theory and Self-Determination Theory. Emotion Processing Theory emphasizes that emotional responses are involved in individuals' appraisal of the value and meaning of a situation, thereby influencing whether they continue to invest effort in a given activity (Lazarus and Folkman, 1984). In training contexts, positive emotional feedback can reinforce athletes' perceived meaning of effort, progress, and challenge. Conversely, emotional blunting may weaken this feedback function, making it more difficult for training activities to provide sustained psychological rewards. Existing research suggests that emotional numbness or blunting may reduce emotional fluctuations in response to activities that are otherwise meaningful, thereby limiting behavioral mobilization (Litz and Gray, 2002; Frewen and Lanius, 2006). From the perspective of Self-Determination Theory, high-quality engagement also depends on athletes' identification with and internalization of training activities. When the emotional connection between training activities and personal goals weakens, voluntary effort and sustained engagement may also decline (Ryan and Deci, 2000, 2017).

These results suggest that emotional blunting may serve as a key proximal indicator of declining training engagement. Compared with fatigue, complaints, or explicit training aversion, emotional blunting is more subtle and therefore more easily overlooked. Athletes may continue to attend training sessions on time, complete assigned tasks, and follow instructions, while their intrinsic engagement has already begun to weaken. Therefore, coaches should not assess athletes' training status solely through training volume, task completion, or overt discipline. They should also attend to signs such as muted responses to training outcomes, reduced excitement about progress, and diminished anticipation of challenging tasks. If these signals persist, training management should adjust feedback methods, task difficulty, and goal explanations to help athletes recover positive emotional feedback and perceived meaning during training.

### Situational heterogeneity and conditional process mechanisms: a moderating explanation of gender and project type

Results from multigroup structural equation modeling indicated that the chain of associations among training control intensity, cognitive exhaustion, emotional blunting, and training engagement was not entirely consistent across groups. Gender primarily moderated the pathway from training control intensity to cognitive exhaustion. The path coefficient was higher among female athletes than among male athletes [β_female = 0.60; β_male = 0.51; Δχ^2^(1) = 4.49, *p* = 0.03]. This finding suggests that, under comparable levels of training control, female athletes may be more likely to report higher cognitive exhaustion. It should be emphasized that this result does not imply lower resilience among female athletes. Rather, it indicates that the psychological resource costs associated with training control intensity may vary across gender groups. Continuous self-calibration demands arising from frequent error correction, external monitoring, and punishment expectations may correspond to different degrees of psychological resource depletion across athletes.

This gender difference can be understood from the perspectives of training experience and self-regulatory load. Self-Determination Theory emphasizes that controlling contexts restrict individuals' autonomy in interpreting and engaging with activities, making behavior more dependent on external demands (Ryan and Deci, 2000, 2017). Self-Regulatory Resource Theory further suggests that sustained performance monitoring, emotional suppression, and behavioral adjustment consume limited psychological resources (Muraven and Baumeister, 2000; Inzlicht and Schmeichel, 2012). In training contexts, if female athletes are more sensitive to evaluative cues, corrective feedback, and interpersonal pressure, controlling training arrangements may be more strongly associated with cognitive exhaustion. Thus, gender in this study is not merely a demographic grouping variable but also reflects potential differences in how the psychological costs of training control are experienced.

The moderating effect of sport type was mainly observed in the middle and later segments of the chain. On the pathway from cognitive exhaustion to emotional blunting, the association was stronger in individual sports than in team sports (β_individual = 0.73; β_team = 0.56). Similarly, on the pathway from emotional blunting to training engagement, the negative association was stronger in individual sports than in team sports (β_individual = −0.54; β_team = −0.39). These findings suggest that athletes in individual sports may be more likely to experience reduced emotional reactivity when cognitive resources are highly depleted. When emotional blunting increases, their decline in training engagement may also be more pronounced. Individual sports often involve more concentrated responsibility attribution and more direct performance evaluation, making training success or failure more closely tied to athletes' personal performance. In this context, reduced emotional responsiveness following cognitive exhaustion may be less easily buffered by team interaction, role sharing, or peer support and may therefore be more closely associated with decreased engagement.

By contrast, shared goals, role differentiation, and peer interaction in team sports may, to some extent, weaken this association. Training engagement among team-sport athletes depends not only on individual emotional mobilization but also on team responsibility, collective rhythm, and interactive feedback. Even when an athlete shows a certain degree of emotional under-reactivity, the team structure may continue to provide external support and behavioral guidance. This may partly attenuate the negative association between emotional blunting and training engagement. This finding is consistent with existing perspectives on athlete burnout, impaired emotional functioning, and sport-specific differences (Gustafsson et al., 2018). In addition, training engagement, as a state involving focus, effort, and persistence, is also shaped by training organization and social interaction structures (Schaufeli et al., 2002).

Overall, the moderating effects of gender and sport type indicate that the psychological pathways embedded in high-control training contexts are subject to specific boundary conditions. Gender differences were mainly reflected in the association between control demands and cognitive exhaustion, whereas sport-type differences were concentrated in the later stages of the pathway, namely from cognitive exhaustion to emotional blunting and from emotional blunting to training engagement. These findings suggest that training management should not rely solely on overall average effects to formulate uniform strategies. Instead, risk identification should consider both group characteristics and sport-specific organizational structures. For female athletes, greater attention should be given to cognitive resource depletion associated with high-control feedback. For athletes in individual sports, early identification of emotional blunting and declining engagement is particularly important. Supportive feedback, meaningful explanation, and appropriate autonomy may help reduce the psychological burden associated with sustained control.

## Conclusions and recommendations

### Conclusions

Based on Self-Determination Theory, Self-Regulatory Resource Theory, and Emotion Processing Theory, this study constructed and tested a chain mediation model linking training control intensity, cognitive exhaustion, emotional blunting, and training engagement. Using cross-sectional questionnaire data, structural equation modeling, and multigroup analysis, the study reached the following conclusions.

1. Training control intensity was significantly and positively associated with cognitive exhaustion. Athletes who perceived higher levels of training control tended to report greater cognitive exhaustion, suggesting that high-control training contexts are closely related to increased self-regulatory load and psychological resource depletion.
2. Cognitive exhaustion was significantly and positively associated with emotional blunting. Higher levels of cognitive exhaustion were accompanied by reduced emotional reactivity and a stronger tendency toward emotional flattening. This finding suggests that athletes' psychological resource state may be a key factor in explaining changes in emotional responses within training contexts.
3. Emotional blunting was significantly and negatively associated with training engagement. Athletes with higher levels of emotional blunting, characterized by reduced emotional reactivity and weakened emotional feedback, tended to report lower levels of training focus, proactive effort, and sustained engagement. This finding indicates that emotional blunting may serve as an important proximal psychological indicator of declining training engagement.
4. Cognitive exhaustion and emotional blunting formed a significant chained mediating pathway between training control intensity and training engagement. This result supports the theoretical pathway of resource depletion → emotional deactivation → reduced engagement. However, because this study used a cross-sectional design, the chained mediation effect should be interpreted as a statistically significant indirect association rather than as evidence of a strict causal sequence.
5. Multigroup analyses showed that this relational structure differed across gender and sport type. Gender primarily moderated the pathway from training control intensity to cognitive exhaustion, whereas sport type mainly moderated the pathways from cognitive exhaustion to emotional blunting and from emotional blunting to training engagement. These findings indicate that the strength of the proposed psychological process may be jointly shaped by individual characteristics and training-context factors.

### Recommendations

Based on the above conclusions, this paper suggests that training management should reconceptualize “control” not merely as a disciplinary tool or means of enforcement, but as an adjustable contextual variable within the resources–emotions–engagement mechanism. Greater attention should be given to developing clear, actionable, and traceable institutional arrangements for communication rules, feedback procedures, and control intensity decisions. Such arrangements may help reduce the sustained consumption of athletes' cognitive resources under high-control conditions, prevent cognitive exhaustion from developing into emotional blunting, and maintain high-quality training engagement without weakening training standards. Specific recommendations are as follows.

First, coaches should regulate the frequency of authoritative demands, repeated corrections, and punitive feedback. Clear requirements should be maintained in critical areas such as technical safety and training discipline, while athletes should be given appropriate room for interpretation and adjustment during exploratory or adaptive training tasks.

Second, training feedback should be specific, concise, and actionable. Coaches should reduce continuous and generalized negative evaluations and instead adopt a structured feedback approach that follows the sequence of problem identification, cause explanation, and direction for improvement. This approach may reduce the psychological burden associated with athletes' constant self-monitoring.

Third, training management should monitor signs of cognitive exhaustion and emotional blunting, including post-training concentration difficulties, slowed responses, limited emotional reactions to training outcomes, and diminished anticipation of challenging tasks. These signs should be treated as important indicators for adjusting control intensity, training load, and feedback methods.

Fourth, greater attention should be paid to female athletes and athletes in individual sports, as they may be more vulnerable to psychological strain and emotional blunting under high-control training conditions. Coaches can help these athletes sustain training engagement by increasing communication transparency, providing more supportive feedback, and strengthening the perceived meaning of training goals.

## Limitations

Although this study provides empirical evidence on the relationships among cognitive exhaustion, emotional blunting, and training engagement in high-control training contexts, several limitations should be acknowledged. First, the study used single-time-point, cross-sectional self-report data. Structural equation modeling and chained mediation analysis mainly reveal statistical associations and theoretical model fit among variables. They cannot establish the temporal ordering of variables or support strict causal inferences. Future research could employ longitudinal tracking, cross-lagged designs, diary methods, or experimental interventions to further examine the dynamic relationships among training control intensity, cognitive exhaustion, emotional blunting, and training engagement.

Second, although this study addressed potential common method bias through procedural controls, Harman's single-factor test, and one-factor confirmatory factor analysis, the core variables were still primarily measured using athlete self-report questionnaires. The results may therefore remain susceptible to social desirability bias and transient emotional states. Future studies could incorporate coach evaluations, training observations, objective load records, and behavioral indicators of engagement to strengthen multi-source data validation.

Third, the sample was drawn mainly from China's university competitive training programs and provincial training units. These contexts are characterized by hierarchical management, centralized training arrangements, authoritative coaching structures, and performance-oriented evaluation systems. Therefore, the findings are more applicable to standardized competitive training contexts in China. Their generalizability to other countries, professional clubs, community sports organizations, or youth development systems requires further examination. Future research could conduct comparative studies across cultural settings, governance models, and training organizational structures to clarify the boundary conditions of the proposed chain of associations.

Finally, this study examined only the moderating effects of gender and sport type. Other potentially relevant factors, such as coaching leadership style, team climate, training experience, injury history, and competition cycle, were not fully incorporated into the model. Future research could extend the current framework by including additional contextual and individual variables, thereby providing a more comprehensive explanation of athletes' psychological adaptation in high-control training environments.

## Data Availability

The original contributions presented in the study are included in the article/supplementary material, further inquiries can be directed to the corresponding author.
